# Patient Expectations: Searching Websites on How to Apply to Access Medical Records

**DOI:** 10.3390/ijerph19116503

**Published:** 2022-05-26

**Authors:** Kay Nicol, Kim Lehman, Joan Carlini, Kathleen Tori, Kerryn Butler-Henderson

**Affiliations:** 1 College of Health and Medicine, University of Tasmania, Hobart, TAS 7005, Australia; kerryn.butler-henderson@rmit.edu.au; 2 Liverpool Business School, Liverpool John Moores University, Merseyside L3 5UX, UK; k.f.lehman@ljmu.ac.uk; 3 Department of Marketing, Griffith University, Nathan, QLD 4111, Australia; j.carlini@griffith.edu.au; 4 Faculty of Health and Education, Torrens University Australia, Melbourne, VIC 3000, Australia; kathleen.tori@torrens.edu.au; 5 School of Health and Biomedical Sciences, RMIT University, Melbourne, VIC 3000, Australia

**Keywords:** medical records, access, websites, expectations, expectation confirmation theory

## Abstract

Patients who want to know how to access their medical records from a health organization’s website have certain expectations about what must be included to assist in this process. The purpose of this article is to detail patient expectations of a health care organization website when searching for information on how to apply for access to their medical records. Using expectation confirmation theory, a survey was developed to ask patients, as consumers of health care, about their expectations when accessing websites. The results revealed that patients want websites to be safe and secure and have help available if there are questions about the website or search functionality. In order to improve the patient experience, health care providers need to understand these expectations from the patient perspective about this information-seeking exercise.

## 1. Introduction

It is well documented that patient-centered care includes providing knowledge and access to information about health care to assist patients in making informed decisions about treatment and enhancing communication with providers [1,2,3]. The increased use of information technology and access to the internet has given patients, as consumers of health care, the ability to search online to access a range of goods and services, including websites for general health information [4]. A recent systematic review highlighted that patients are not aware of the process to apply to access their own information (i.e., medical records) [5]. Furthermore, patients are interested in accessing their records, but over one-third did not know they had the legal right to do so [6].

There is a plethora of literature about patient access to medical records via personal health records, electronic medical records, and online portals, and about the impact of increased digitization on access for both patients and health care organizations [7,8]. However, what if there is not an online portal? How can patients find information about the process of requesting access their medical records? Most organizations still require patients to telephone their medical record department to ask about the process, yet consumers today want to avoid the phone call if they can instead find information about a process online. There is no literature examining patient expectations about what information should be available on websites about the process of requesting access to a medical record [5,7,9].

This distinct lack of empirical evidence in what information patients expect to find on a website about how to apply for their own health information provided an opportunity to address this dearth of knowledge. Allowing patients to directly inform health care organizations of exactly what they expect to find and what information they need facilitates consumer input into websites, which ultimately enhances the patient experience and satisfaction [3,10]. This research was undertaken to address the lack of knowledge about patient expectations and to provide unmistakable evidence of exactly what patients want to enable their experience to be improved. The aim of this paper is to present patient expectations of information on health care organization websites about how they can apply to access their medical record.

## 2. Materials and Methods

This research used a survey underpinned by expectation confirmation theory (ECT) to collect the data. Broadly, ECT proposes that consumer satisfaction with a product or service is determined by the interaction between prior expectations and the perceptions of delivery [11]. There are four components of the theory: (A) expectations; (B) performance; (C) a comparison of expectations versus performance; and (D) satisfaction [12,13]. ECT has been used for marketing research [14] and applied in other disciplines including tourism [15], retail [16], computers and information technology [14,17,18], and web services [19,20]. Considering the theory’s broad application, it was deemed an ideal theoretical framework to investigate the problem at hand. This paper specifically concerns component (A) above, which is “expectations”. Respondents were asked what they would expect to find on a website of a health care organization if they were seeking information on how to apply to gain access to their medical records.

This survey was based on results from two empirical studies that used ECT [21,22] and was validated by the research team [5]. The survey included several five-point Likert-scale questions concerned with expectations of searching for information on websites. First, a level of agreement with statements about information one might expect to find relating to this process was sought. Second, a level of importance was required in response to a list of expected characteristics. Lastly, the section included an open-ended question, “What other aspects of a health care organization’s web page, in your opinion, do you consider important when searching for information on how to apply to access your medical records?” to gain greater insight into the perceptions, expectations and opinions of people who seek this information. These survey questions are presented in Table 1.

The study used a convenience sample. Eligibility criteria required consenting participants to be aged 18 years or over, had experienced a visit to a health care organization in either Australia or the United States of America (US) and be residing in either of these countries at the time of completing the survey. Access to the internet was required, as the survey was only offered online. Participants in Australia and the US completed the survey through the crowdsourcing platform, Amazon’s Mechanical Turk (MTurk).

The survey was built in the research software program REDCap^®^ (Research Electronic Data Capture) version 8.5.19, (Hobart, Australia), and then launched on MTurk in April 2020. Each opened survey was allocated a participant identity number in REDCap^®^, and no identifiable data were recorded. The survey was available online for eight weeks until the maximum number of completed surveys was reached, and then quality checked. Once finalized, the responses were exported from REDCap^®^ to Microsoft Excel (2016) and the data were cleaned. Findings from the qualitative and quantitative data of the survey explored the richness of information provided from the perspective of the patient as a consumer of health care, which can inform health care organizations about this important issue. Specifically, it draws on both descriptive statistics from Likert-scale questions and a qualitative analysis of responses to the open-ended question. Qualitative data analysis of the open-ended question responses was performed using NVivo 12 Pro [23], which allowed the research team to create and organize themes hierarchically. These responses were thematically analyzed by authors 1 and 2 using an inductive approach [24,25], in which comments were interrogated to produce the preliminary first round coding. Following a synthesis process to detect any overarching themes, whereby common concerns and issues were grouped together, and discussion and agreement with the research team, the final themes were produced.

## 3. Results

**Figure 1:**
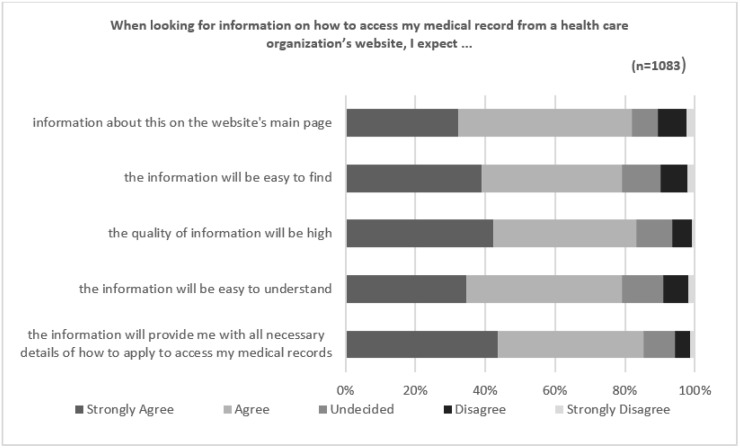
Expectations of health care organization websites.

**Figure 2:**
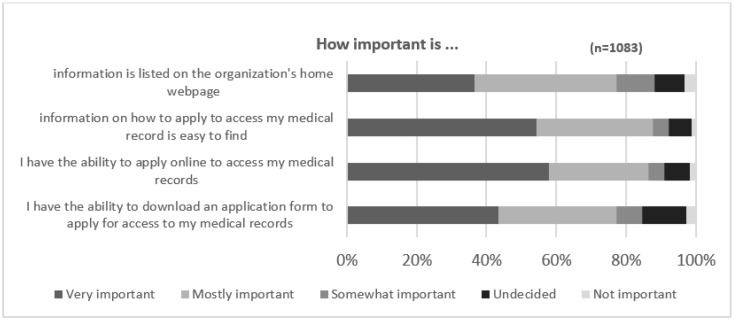
Importance of information on health care websites.

A total of 1725 respondents completed the section of the survey being analyzed in this paper. Of those, 1083 were deemed valid responses, of which 62 (5.7%) were from Australia and 1021 (94.3%) were from the US. Known demographic information provided by 509 (47%) responses included 238 (46.76%) female, 265 (52.06%) male and 6 (1.18%) non-binary. Only comprehensible responses to the open-ended question were included. Responses such as “not applicable”, “nothing to add”, “none” and statements copied and pasted from the survey questions were excluded from the analysis, as these did not add meaning to the results. A summary of the responses to the statements preceding the open-ended question is provided in Figure 1 and Figure 2 below.

### 3.1. Quantitative Responses: Descriptive Statistics

#### 3.1.1. Website Expectations

To gauge patient’s expectations of what might be included on a health care organization’s website, respondents were asked to rate their level of agreement to five statements (see Figure 1).

A considerable proportion of respondents (82%) expected to find information about the process on the website’s main page. Similarly, respondents expected that the information provided would include all the necessary details required to make an application to access records (85%), and 83% expected the quality of the information to be high. More than three quarters (79%) expected that the information would be easy to find and easy to understand.

Respondents who strongly disagreed, disagreed or were undecided (18% average) with any of the expectations at that point of the survey possibly suggested either (a) no experience or preconceived expectations of a health care website or (b) previous experience and found websites lacking in these characteristics.

#### 3.1.2. Previous Experience Searching Websites

Slightly more than half of the respondents (55%) had experience in searching a health care organization’s website on the process of how to apply to gain access to their medical records. Despite almost half of the respondents (45%) having no experience, expectations of those seeking this type of information were rated high (82% average across all given expectations), suggesting a certain level of importance placed on this process.

#### 3.1.3. Information on Websites

The importance of easily finding information on how to apply for access to medical records was shown to be very important or mostly important (88%). Similarly, the ability to be able to download an application form to apply from the website (87%), the ability to apply online (86%), and the placement of the process on the organization’s home webpage (77%) were all rated very important or mostly important (see Figure 2). One in ten respondents (11%) rated the information being found on the home page of a website to be somewhat important. Although consumers consider information on these processes as important, they are not expecting it to be visible on the landing page of a website and are willing to search for this.

### 3.2. Qualitative Responses: Thematic Analysis

This first round coding and thematic analysis resulted in the following themes: (1) security and privacy; (2) accessibility to records, incorporating the ease of finding the information; (3) the requirements for the process and the length of time to obtain the records; (4) contact details and available help; (5) website design; (6) accuracy and credibility; and (7) the provision of medical information including links to test results, appointments, and medical advice. The definitions assigned to these themes, as well as examples, are provided in Table 2.

Following the second round of coding, the following three overarching themes were apparent: “ease of use”, “breadth of services”, and “privacy of information”. The three overarching themes are discussed below, with examples provided by the respondents shown in italics.

#### 3.2.1. Ease of Use

The data revealed that the overarching theme, “ease of use”, encompasses all those factors that concern the process of finding and accessing information, and the process aligns with consumer expectations of a professional health care provider.

The first factor is accessibility of the information, which includes the types of information consumers are permitted or able to access. Generally, the respondents indicated that they are prepared for some items not to be available and for there to be potential costs, but they want to know up front:

*I should be given information on all potential ways I can access my medical records. I should also be given a sense of the time it’ll take (especially if I have to apply by mail), and also what information will be available.*

*I want to know information regarding any charges applied in accessing my medical records.*

The design of the website, as the interface between the consumer and the organization, is crucial. At its most basic, consumers want the organization’s website:

*… to be user friendly and make it easy to find what I am looking for by allowing me to enter what I want into a search box. They should clearly state what is required and what I will need to access my medical records. They should state if there are any extra steps that I might have to take before gaining access to them.*

Respondents wanted the website design to “be usable and not clunky”. Phrases such as “easy to navigate” and “easy to use” were consistently seen, as were words such as “functionality”, “logical”, “modern” and “intuitive” appeared regularly. These responses indicate that consumers have a high level of expectation when it comes to website design and consumer interfaces.

Within the overall design requirements, respondents expected to have their access point clearly marked:

*It needs to be on the first page I see and very clear to see. If someone is accessing their records, they shouldn’t make it hard for someone to get their rights.*

Some respondents had quite high expectations of the website in this respect:

*I expect to see a fairly conspicuous patient portal link for access of medical records. As we now have online medical records, I expect to be able to access my medical records directly through the patient portal on the provider’s website and not require an application form. I should not need to ’apply’ to access my own records.*

The ramifications for not getting this right are not only disgruntled consumers but consumers with no faith in the online systems:

*It must be easy to sign up and register for using the website. I had a bad experience after a medical visit, I tried to sign up and it never let me in, I finally just gave up and left with a bad impression of medical record usage online.*

#### 3.2.2. Breadth of Services

The overarching theme “breadth of services” revealed several factors consumers expected when communicating and engaging with providers of health care when using a website. These ranged from being able to ask for help when requesting information to knowledge about the professionals listed by the provider and the types of services offered.

Overwhelmingly, the responses centered around the provision of help with the online request including details of who to contact, how to make contact and the method of communication. Many respondents required a phone number to call, the ability to send a message or an online chat to help with requests:

*I consider the inclusion of a FAQ section and a phone number that I could call for support to be important when I am searching for the info on applying to access my medical records.*

*It would be nice to have a chat window or help desk number or a least an email to contact someone for help accessing the information.*

Phrases such as “I look for a phone number as well or some form of contact”, and words such as “contact information”, “help and support”, “phone number”, “email”, “customer support” appeared regularly in the responses. This indicates consumers have high levels of expectations about accessible help when trying to process these types of requests.

Another aspect of this theme was the expectation that information about the professionals at the organization and the services on offer:

*I like the staff references section. This allows me to conduct research using reviews about medical staff. I usually like to assess a large volume of reviews before I choose a medical professional and/or office for my healthcare needs.*

*Listing of their doctors and care providers along with their specialty and what services they offer.*

Having this type of knowledge provides consumers with information for informed choices about care and treatment options. Responses also suggested that consumers are interested in viewing services with the ability to book these directly online:

*Quality of services offered, how to access them.*

*Easy to access information about office visits by dates, diagnosis, test results, future appointments.*

*To schedule an appointment online without having to call the office.*

Several respondents expressed interest in medical information contained in their records, such as medical terminology, information about medicines, diagnoses, and diseases:

*I hope to see information that will help me to understand what I am reading in regards to my medical records.*

*Ease of learning what the information given means. Medical information can be hard to read.*

*FAQs about medical records and if there are any questions that you may have about deciphering them or accessing them or sending them along to other medical professionals.*

An additional aspect of this theme was that consumers also expect information stated on the website to be accurate and up to date. Expectations of accuracy referred to the accuracy of the information the health care organization kept about the consumer, which highlighted the complexity of personal health information being stored across many organizations for a single consumer:

*Other important aspects would be signs or indications that all info presented is up to date and accurate.*

*They should tell me how complete the records will be considering if I have recently changed doctors.*

#### 3.2.3. Privacy of Information

The theme “privacy of information” incorporates two major areas—privacy and security. Not surprisingly, among all the topics surveyed, the areas of privacy and security were of most concern to respondents. The data clearly revealed the high levels of expectations required to protect the privacy and confidentiality of one’s health records and the assurances essential for the security of the website when applying for access to this information.

Safety and security of the website were featured most frequently in the responses as well as words and phrases such as “safety and security”, “privacy, making sure no unauthorized person will have access to my data”, “my information is secure”, “guarantee of privacy”, “data security”:

*They should have some sort of process that will verify my information so as to protect my medical record information, and make sure only I am able to access it (besides the organization itself).*

*I would hope that confidentiality would be a top concern of the medical organization, and that it would thoroughly ensure I am who I say I am when attempting to apply to access my medical information.*

While consumers expect health information to be as accessible as possible, importance is placed very highly on the website being sufficiently secure to handle this type of interaction online, including those strategies being publicly available:

*I would like an explanation for how my data is kept safe online and what might be done in the event the data is accessed inappropriately.*

*The information is behind a firewall and/or encrypted to ensure privacy and confidentiality. What’s the site’s current IT security standards and protocols and what software are needed to access, read, and download my information.*

Generally, consumers seemed willing to complete the necessary forms and steps for identity checks to ensure their health records were safe and secure and not accessible by anyone:

*The most important aspect would be that the organization is keeping my records safe and secure. I would want the process to accessing my medical records to be easy however I would want the records to be safe and secured so that others cannot access them.*

*I usually consider how serious they take about releasing my medical records and look at the paperwork that is needed to fill out before accessing the records.*

## 4. Discussion

The purpose of this paper was to present the findings of a survey designed and underpinned by ECT to examine patient expectations of searching online for how to apply to access personal health records. It is well documented in the literature how accessing medical records contributes to patients being better informed about their health care choices, treatment options and enhances communication with the provider [9]. The rise of electronic and digital records and the use of patient portals provide the patient with more access than ever before [9]. Patients need to know how to apply to obtain access to their records, and searching health care organization websites via the internet should provide this direction [10].

The results from this study indicate that patients find it challenging to find information online on how to access their medical records and may need to contact their health care organization directly (face to face, telephone) or indirectly (online search, patient portal). Little empirical evidence is available from the patient perspective of what they want to know about undertaking this process. Asking patients exactly what they expect from health care organization websites considers the patient experience, which is important in knowing exactly what information consumers want and need [26].

Characteristics of website expectations, including all necessary information to be provided and easy to find, easy to understand, high quality and expected to be found on the website’s main page, were rated as highly important. The expectations derived from the inductive analysis resulting in the overarching themes of ease of use, the breadth of services offered, and the aspects of privacy and security of information provided undisputed evidence of patient expectations.

The open-ended question responses were approached in two distinct ways. There were those respondents who carefully considered the requirements of “what information would you expect to find on a website about how to apply for records”. By directly answering the component of initial access and related expectations, it provided valuable insights from the perspective of a patient who wants or needs access and is searching how to do this, potentially for the first time. However, there were responses relating to access of online records, which is a different aspect suggesting that the patient already has some form of access to elements of their health information and what they expect. For example, comments related to downloading and sharing of information suggest the use of online portals to directly access, display, download and share accessible information from a health care provider’s website.

A possible explanation for these different responses may include the country of residence of the respondent and/or experience with certain health care organizations. Many countries have advanced methods of accessing information online, including patient portals that are available for patients to log in and access information from their medical records, including discharge summaries, appointments, pathology, radiology, pharmaceuticals dispensed and reordering of prescriptions [1,9,27]. In the US, for example, access to medical records through the OpenNotes project helps facilitate the process of sharing information between health care providers and patients [1]. In Australia, My Health Record provides a repository for documents, including diagnostic imaging and pathology reports, discharge summaries, prescriptions, and referral letters that may be shared depending on the settings chosen by the record holder [28].

Many countries have legislation that empowers the patient to access their information. Therefore, health organizations need to provide information about the process to enable this empowerment. For example, the “how to” processes should be visible and easy to understand. Patients also want help to be accessible in the form of an online chat function, a contact number of someone they can talk to if they are unsure or require help, a list of frequently asked questions and the name and number of an agency or department to call if an issue needs escalating. Should the expectation of access be easier and in a more direct method? The accessibility issue highlighted that patients want this information to be visible and not hidden away and difficult to find or referenced in a manner not easily understood.

An important aspect for respondents was the design of the website with useful responses suggesting the inclusion of a search function, signs of a secure website, and ease of navigation. The most dominant theme, however, in the qualitative responses was the level of privacy and security expected of the website and of the processes adopted by the health care organizations for access, storage and sharing of one’s personal health records. Although privacy and security were often referred to as one “privacy and security” in many of the responses, they are independent concepts, yet they might be considered synonymous. Security and privacy issues are well documented in the literature concerning electronic health records [29,30,31,32]. Privacy principles are concerned with how personal information is collected, stored, used and shared [33].

An Australian study regarding community attitudes to privacy revealed that privacy was a major concern for 70% of Australians, and more people are likely to trust a website or service having read the privacy policy [34]. The survey also found one of the biggest risks to the concept of privacy in relation to personal data was security/data breaches, at 61% [34]. The principles of security relate to terms such as misuse, interference, loss, unauthorized access, modification or disclosure of personal information [33].

It was evident from the analysis of expectations in this research that the main expectations of patients undertaking health information-seeking activities are websites that are easy to navigate, with evidence of security and assurances about the privacy of their information. The definitive expectations learned from this research about patient expectations of these processes that have not been investigated before can provide health care organizations the necessary information to improve their websites to assist patients in this crucial step.

### Limitation in the Study

The main limitation of this phase of the research project was the difficulty in reaching the required number of responses from an Australian population, hence the survey being extended to US residents. However, this limitation provided an opportunity to explore the expectations of patients from a more global perspective, confirming that access to personal health information is important regardless of geography. In addition, as this exploratory study was conducted using a convenience sample, future research is required to examine this topic with a more representative sample to be generalized to Australia and US populations.

## 5. Conclusions

This paper examines patient expectations of information on a health care organization website on how to apply to access their medical records. Patients want to locate the necessary information on the main website page; be easy to navigate and understand; and provide a level of assistance if, and when, required. Privacy and security of information; ease of use; and breadth of services are the three key themes derived from the comments and opinions of the survey respondents. Privacy and security of information relate to the security of the website, and privacy relates to the way in which patient information is verified and kept safe ensuring no unauthorized access. Ease of use of the website was expected and is important to respondents and refers to design and functionality of the website. The breadth of services expected on the website include details of the services provided by the health care organization and its professional staff and support when requesting information and answers to frequently asked questions.

Websites are important to convey information about health, and their use helps patients to form expectations and assessment of services [10]. A study using ECT to examine website quality confirmed that factors such as the quality of the information and presentation of the website contribute to consumer knowledge for informed decisions [19]. The expectations of patients as to how they find this information on a health care provider’s website is what makes this research unique and contributes to knowledge in this area.

To empower patients and improve their experience in seeking this information, health care organization providers must ensure that website processes offer patients an easy, safe and secure way to apply to access their medical records. With consumers wanting greater access and control of their health care and in turn health information, organizations have a social responsibility to facilitate this information empowerment of their patients. As such, the findings from this study highlight the need to make the process of how to access their medical records more transparent through their greatest communication tool: their websites.

The next phase in the research project is to analyze the remainder of the consumer survey and use the findings from this phase of the project as the basis to compare expectations with performance and satisfaction of health care organization websites. Future research will include a normative framework to improve websites for patients.

## Figures and Tables

**Table 1:** Survey questions about expectations of health care organization websites.

Scenario: Imagine you are looking for information on how to access your medical record from a health care organization’s website
1. Rate how strongly you agree or disagree with the following statements relating to information on how to access your medical records (five-point Likert scale –”strongly agree”, “agree”, “undecided”, “disagree”, “strongly disagree”)
E1. I expect to see information about this on the website’s main page
E2. I expect the information will be easy to find
E3. I expect the quality of information will be high
E4. I expect the information will be easy to understand
E5. I expect the information will provide me with all necessary details of how to apply to access my medical records
2. Have you ever searched a health care organization’s website before on how to apply for access to your medical records? Yes/No
3. Indicate how important each of the following statements are to you if searched for information on how to access your medical records (five-point Likert scale –”very important”, “mostly important”, “undecided”, “somewhat important”, “not important”)
S1. Information is listed on the organization’s home webpage
S2. Information on how to apply to access my medical record is easy to find
S3. I have the ability to apply online to access my medical records
S4. I have the ability to download an application form to apply for access to my medical records

**Table 2:** Thematic analysis: first round coding rules and themes.

Themes	Definition	Examples
(1) Security, privacy	User makes direct/indirect reference to concerns about security measures required to access records, information on privacy policy, how the records are used and who has access to them, and security of the website.	Security passwords, data encryption, identity verification, the organization’s approach to maintain security and privacy
(2) Accessibility to records, including easy to find the information	User makes direct/indirect reference to the ability to access record, what format will the records take, information should be easy to find and not complicated	Ability to create an account on the site for future access, ability to download and share with other providers without having to complete forms, completely online
(3) Requirements for the process, the length of time to obtain the records and costs	User makes direct/indirect reference to what is required to get access, how long will it take, and how much will it cost to access, if any.	Provided adequate information on all potential methods of accessing records, turnaround times from acknowledgement of request to receiving records, any applicable fees, or charges to obtain
(4) Contact details and available help	User makes direct/indirect reference to contact details if help is needed to find the information and contact details of the medical professionals at the practice	Inclusion of an online chat function, a frequently asked question (FAQ) section, contact details or the ability to speak to a person for assistance, names of practitioners
(5) Website design	User makes direct/indirect reference to the design of the website, easy to read with instructions easy to understand, aspects of the visual design helped to search for the required information	Easy to navigate, well designed, visually appealing and helpful, quick links
(6) Accuracy and credibility	User makes direct/indirect reference to concerns and/or observations regarding the accuracy of the information on the website, the credibility of the website and the practitioners listed	Information contained on the website to be up-to-date and accurate, including the practitioners listed and the details of how to access the required information
(7) Provide medical information, links to test results or appointments, medical advice	User makes direct/indirect reference to medical information to be provided on the website, links to test results, medical appointments, bills and insurances	Advice on medical issues, medicines, different terminology used on different websites affected the ability to search for information

## Data Availability

Access to the data can be obtained from the corresponding author.
